# Supplementary figures and images for: Increased biting rate and decreased Wolbachia density in irradiated Aedes mosquitoes
Source: Parasit Vectors. 2022 Feb 24;15:67. doi: 10.1186/s13071-022-05188-9 (PMC8867665; doi:10.1186/s13071-022-05188-9)

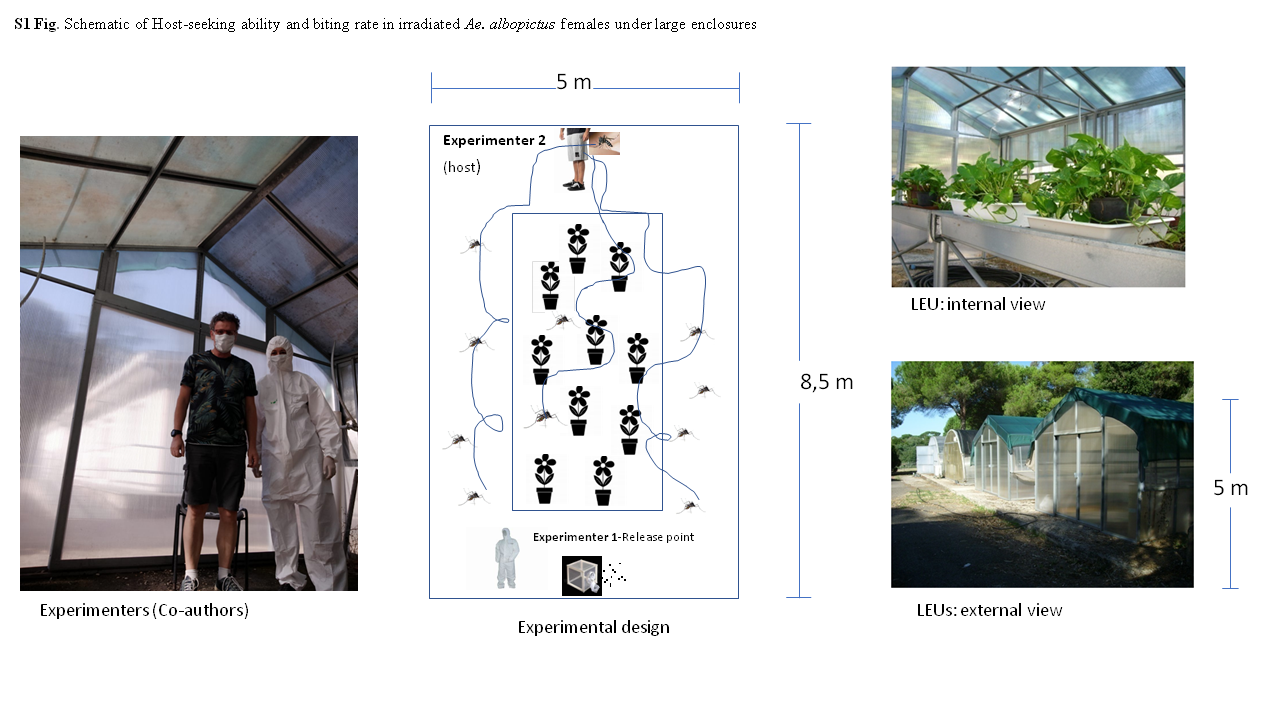

Supplement: Supplementary file 1 — Additional file 1: Figure S1. Schematic of host-seeking behavior trials conducted under large enclosures [file 13071_2022_5188_MOESM1_ESM.tif]

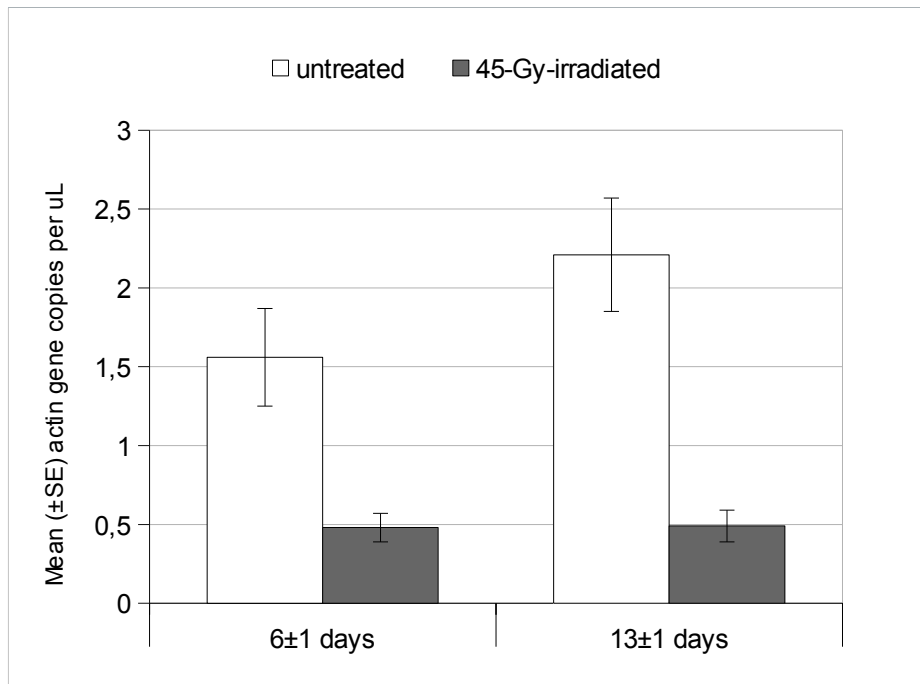

Supplement: Supplementary file 2 — Additional file 2: Figure S2. Actin gene copies in the ovaries of Ae. albopictus females irradiated at 45 Gy in comparison with untreated counterparts [file 13071_2022_5188_MOESM2_ESM.pdf]

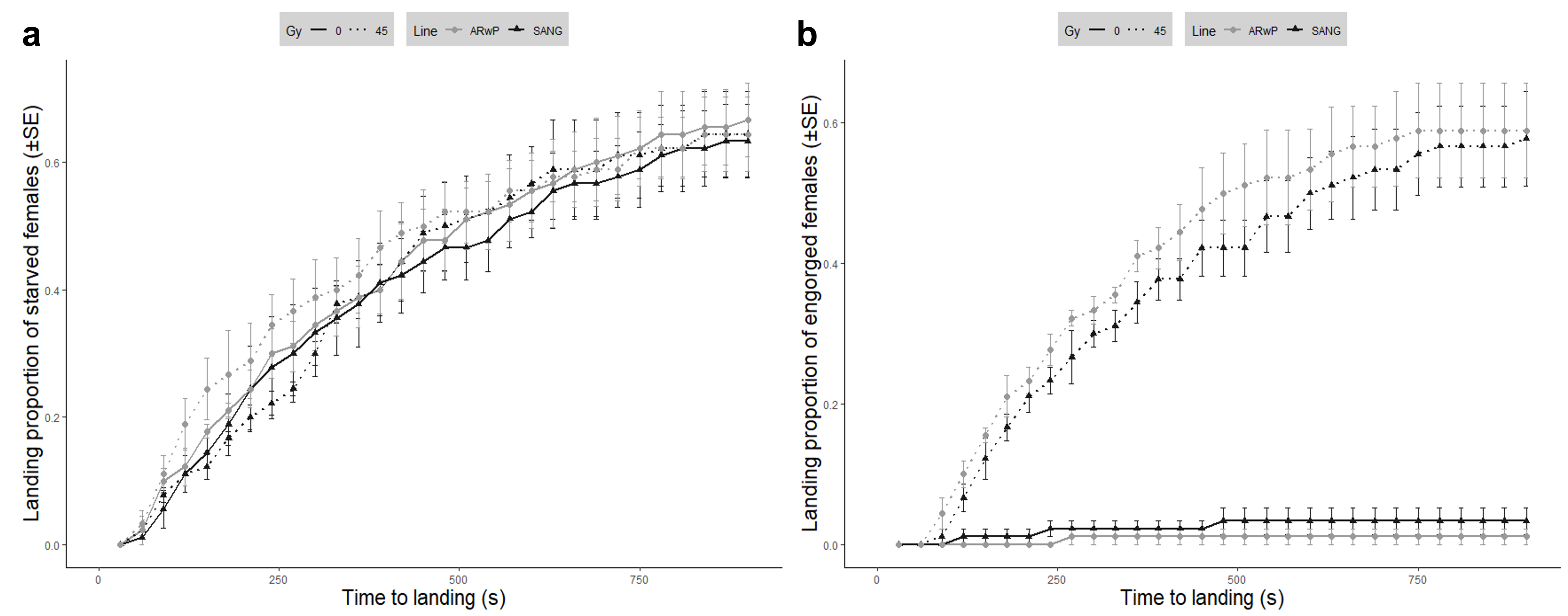

Supplement: Supplementary file 3 — Additional file 3: Figure S3. Host-seeking and biting behavior of irradiated SANG and ARwP Ae. albopictus under large enclosures compared to untreated controls. Biting proportions and average times to landing were compared between treatments within a 15-min interval. a Comparison between untreated and irradiated starved females aged 13 ± 1 days; b comparison between irradiated and untreated engorged females 48 h after the engorgement (i.e., 15 ± 1 days old). Two-way ANOVA demonstrated that the difference between treatments was statistically significant in the case of the engorged females (P < 0.05). [file 13071_2022_5188_MOESM3_ESM.tif]

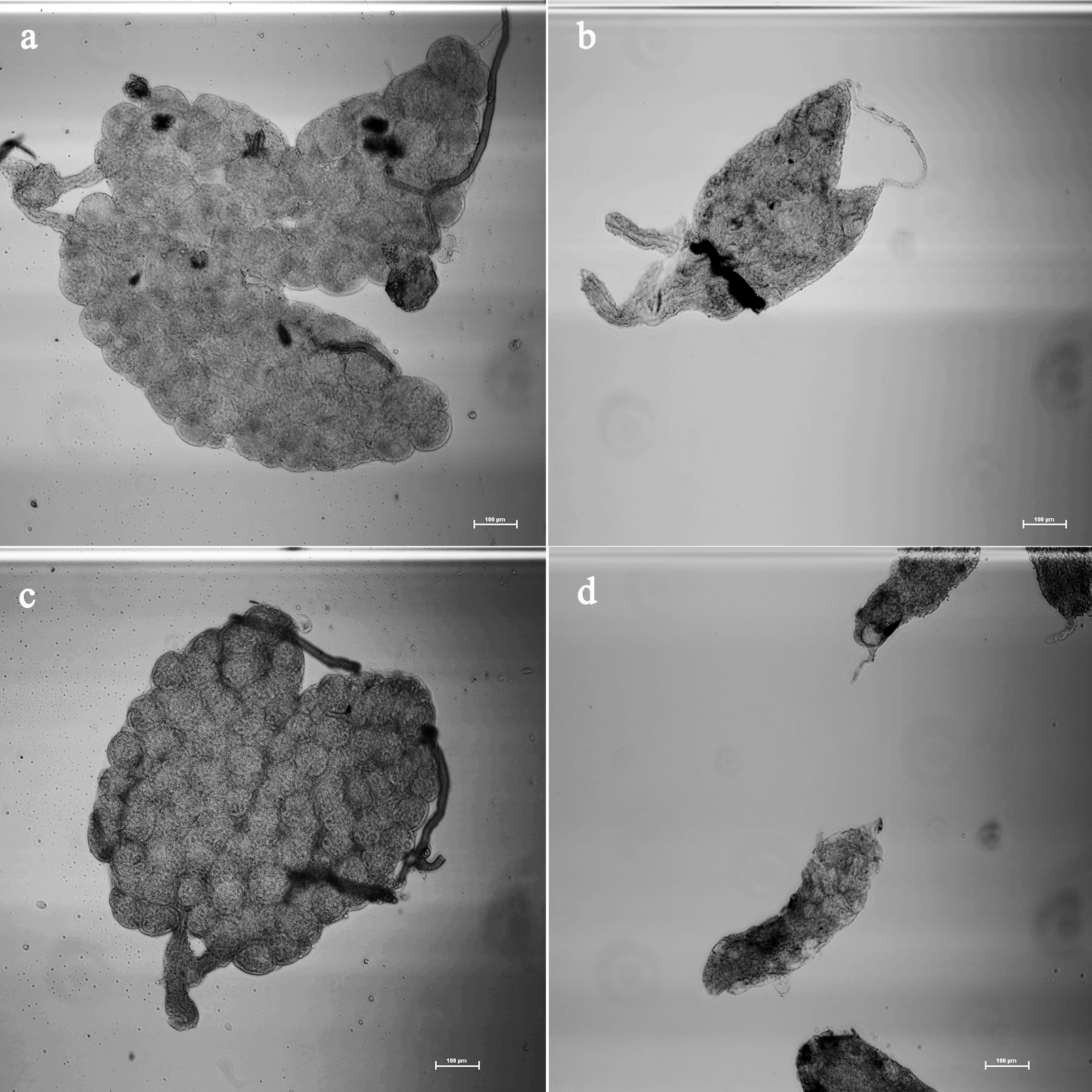

Supplement: Supplementary file 4 — Additional file 4: Figure S4. Structural damage induced by irradiation at 45 Gy in the ovaries of 13 ± 1-day-old Ae. albopictus females in bright field. a Ovaries of untreated SANG females; b ovaries of irradiated SANG females; c ovaries of untreated ARwP females; d ovaries of irradiated ARwP females. [file 13071_2022_5188_MOESM4_ESM.tif]

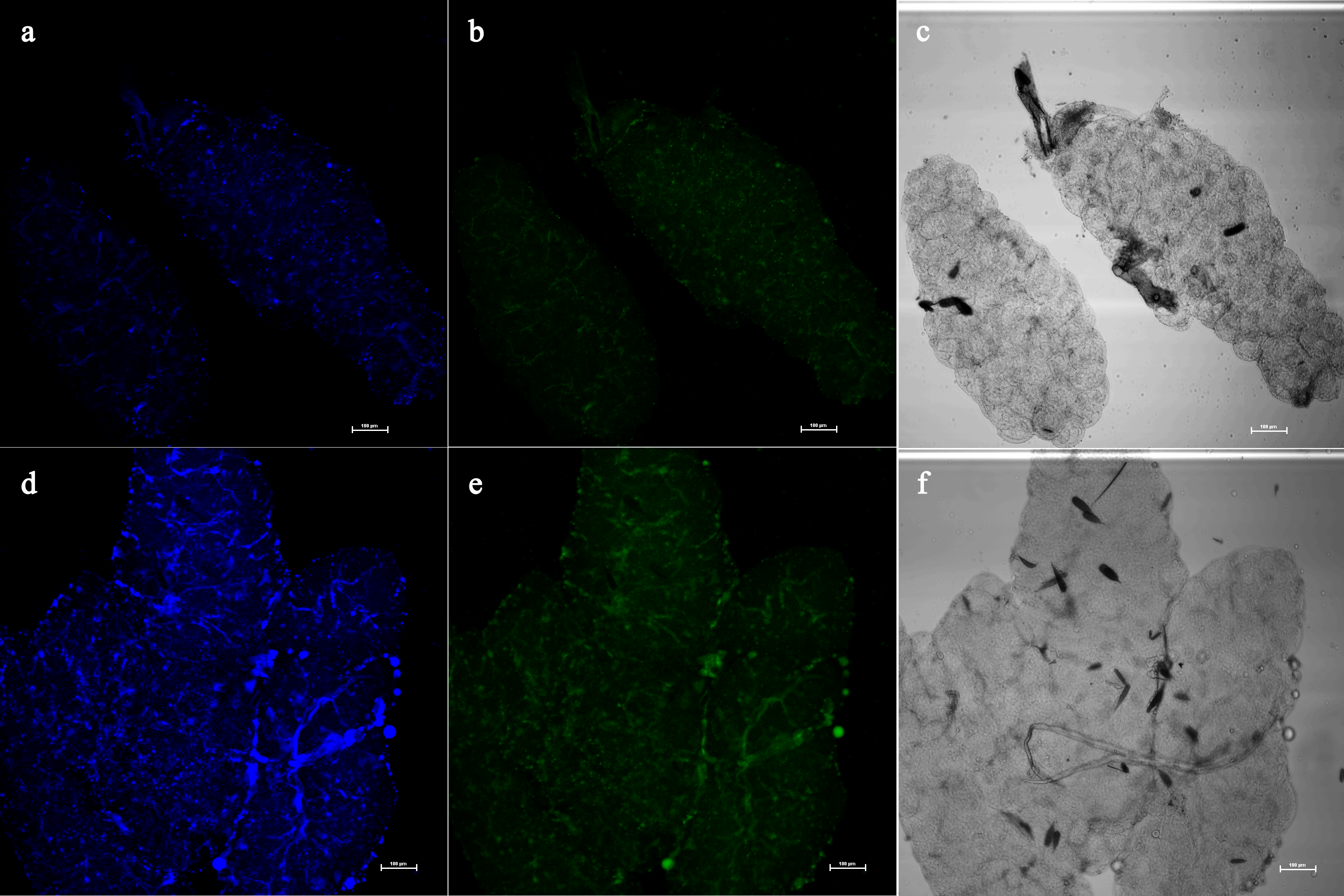

Supplement: Supplementary file 5 — Additional file 5: Figure S5. FISH analysis of the ovaries of 13 ± 1-day-old Ae. albopictus belonging to a line (AR) cured of Wolbachia infection. a and d DAPI-stained; b and e FITC-stained; c and f bright field. No specific green-fluorescent signal was detected in the aposymbiotic line. [file 13071_2022_5188_MOESM5_ESM.tif]

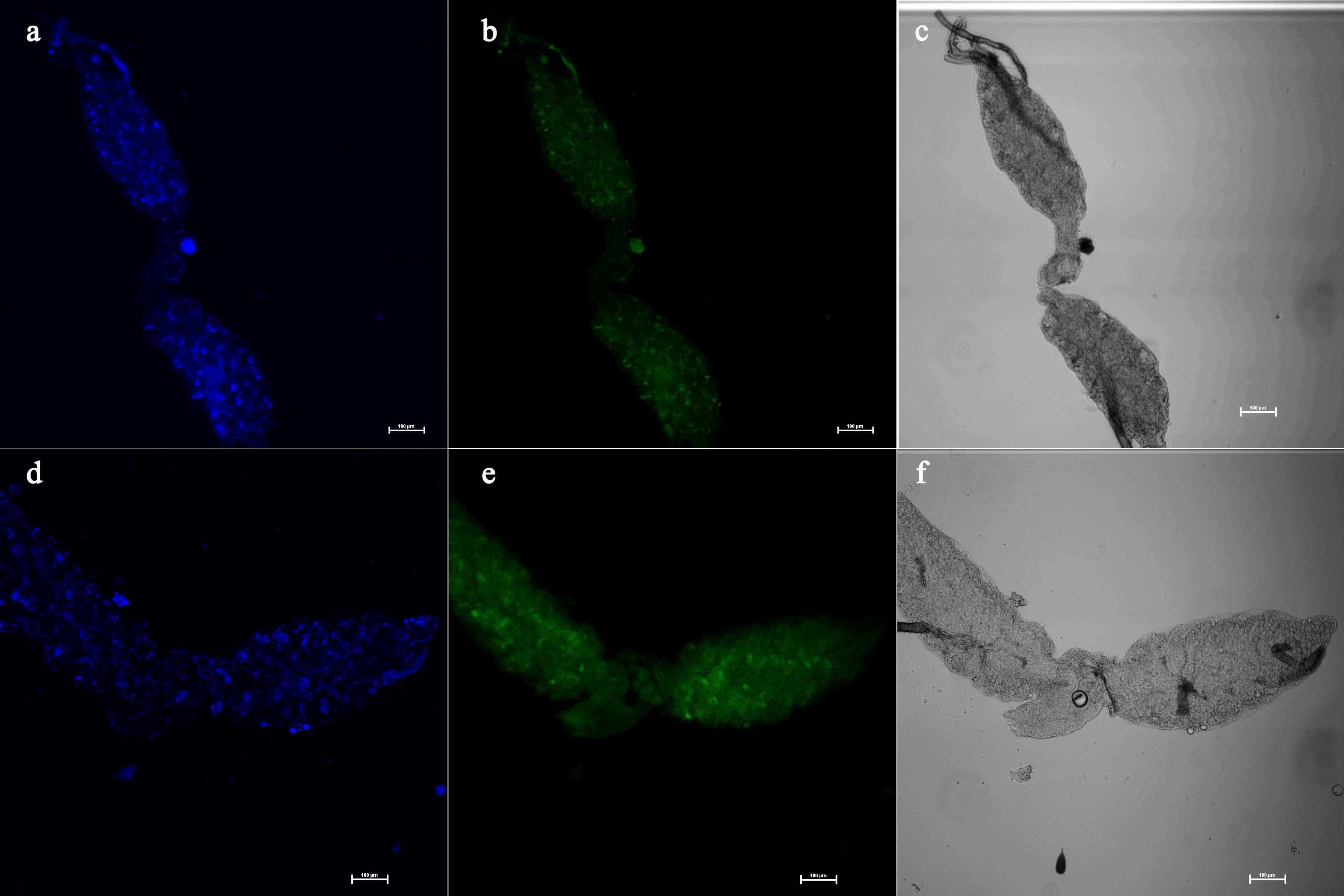

Supplement: Supplementary file 6 — Additional file 6: Figure S6. Additional images related to the FISH analysis of the ovaries of 13 ± 1-day-old SANG Ae. albopictus irradiated at 45 Gy. The distribution of Wolbachia is evidenced in green, while the blue stain is DAPI. a and d DAPI-stained; b and e FITC-stained; c and f bright field. The green-fluorescent signal related to Wolbachia is weak and not homogeneously distributed. [file 13071_2022_5188_MOESM6_ESM.tif]

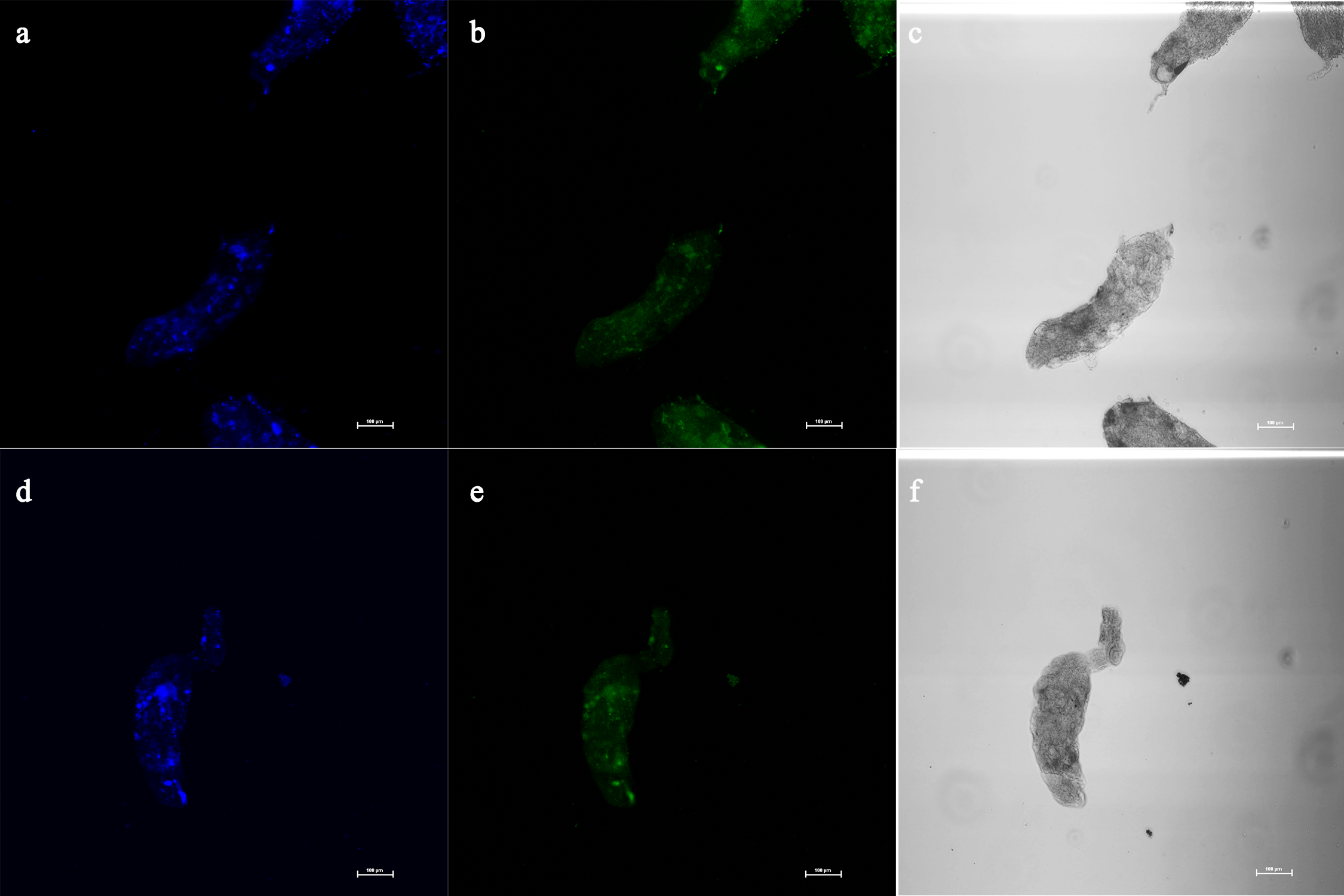

Supplement: Supplementary file 7 — Additional file 7: Figure S7. Additional images related to the FISH analysis of the ovaries of 13 ± 1-day-old ARwP Ae. albopictus irradiated at 45 Gy. The distribution of Wolbachia is evidenced in green, while the blue stain is DAPI. a and d DAPI-stained; b and e FITC-stained; c and f bright field. The green-fluorescent signal related to Wolbachia is weak and not homogeneously distributed. [file 13071_2022_5188_MOESM7_ESM.tif]

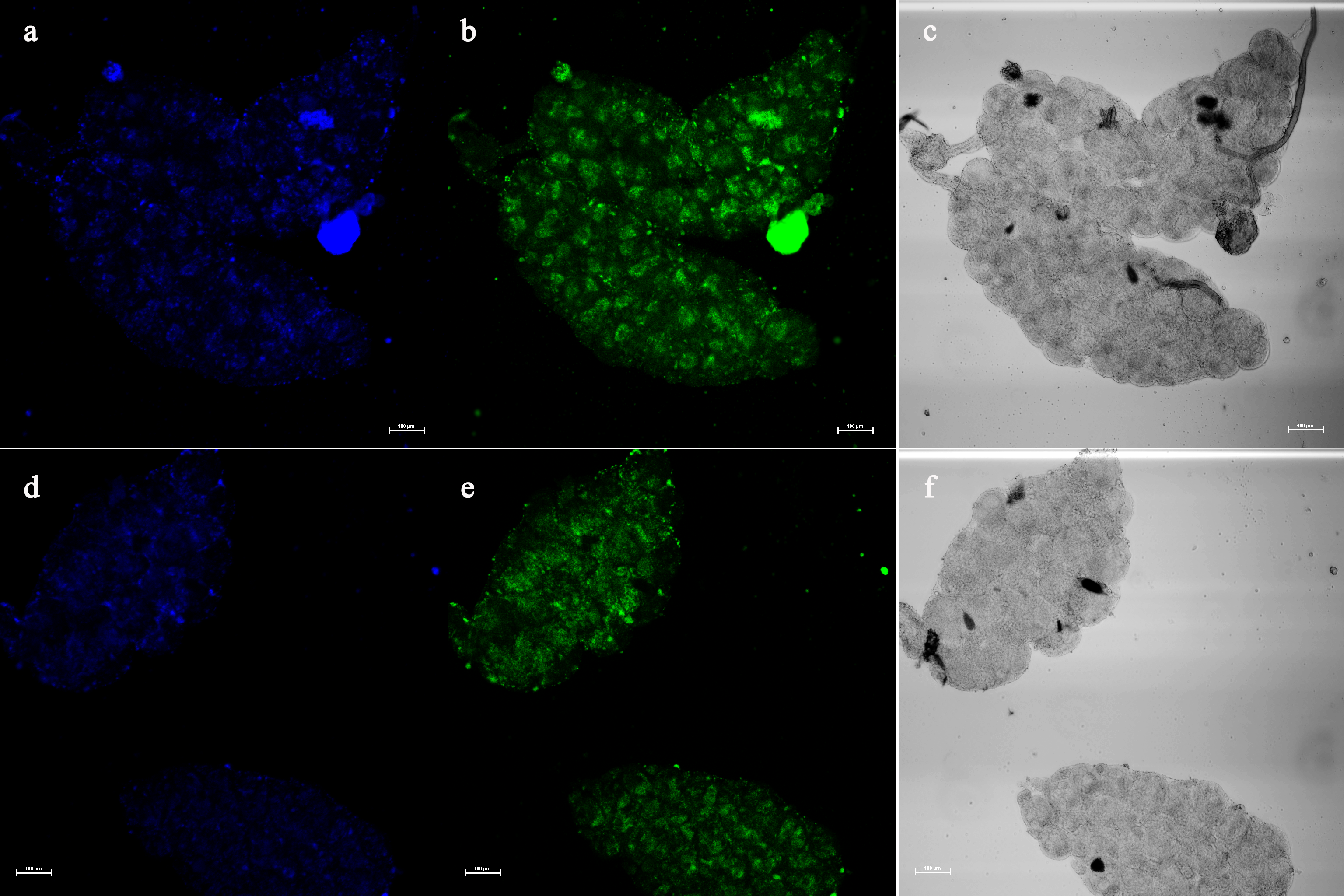

Supplement: Supplementary file 8 — Additional file 8: Figure S8. Additional images related to the FISH analysis of the ovaries of 13 ± 1-day-old untreated ARwP Ae. albopictus. Blue stain is DAPI. a and d DAPI-stained; b and e FITC-stained; c and f bright field. The green-fluorescent signal related to Wolbachia is strong and regularly distributed. [file 13071_2022_5188_MOESM8_ESM.tif]

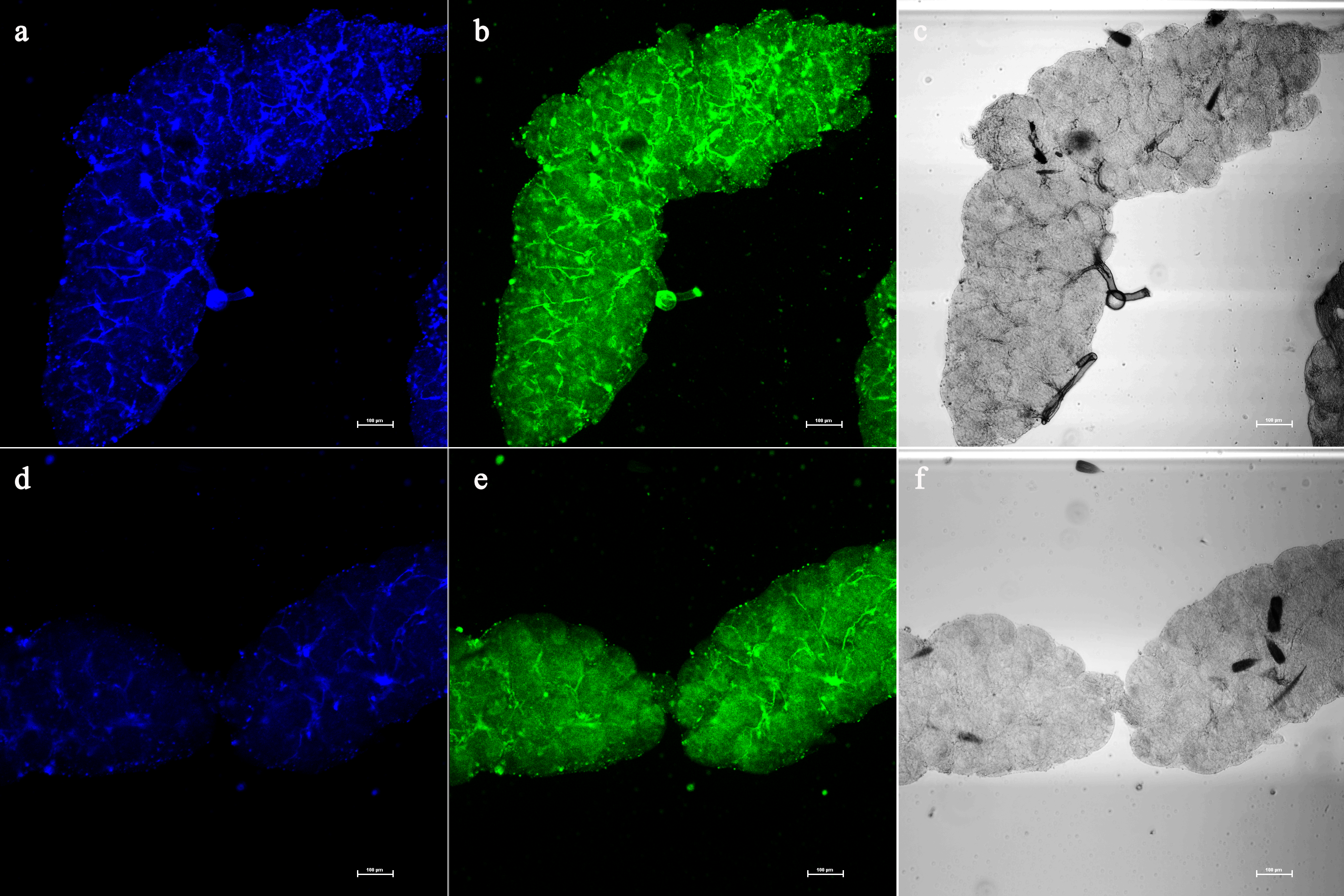

Supplement: Supplementary file 9 — Additional file 9: Figure S9. Additional images related to the FISH analysis of the ovaries of 13 ± 1-day-old untreated SANG Ae. albopictus. Blue stain is DAPI. a and d DAPI-stained; b and e FITC-stained; c and f bright field. The green-fluorescent signal related to Wolbachia is strong and regularly distributed. [file 13071_2022_5188_MOESM9_ESM.tif]
